# Activation of Lymphocytes in Healthy Neonates Within Hours of Birth

**DOI:** 10.3389/fimmu.2022.883933

**Published:** 2022-05-31

**Authors:** Gaayathri Ariyakumar, Sarah Gee, Abhishek Das, Shraddha Kamdar, Rachel M. Tribe, Deena L. Gibbons

**Affiliations:** ^1^ Peter Gorer Department of Immunobiology, School of Immunology and Microbial Sciences, King’s College London, Guy’s Hospital, London, United Kingdom; ^2^ Department of Women and Children’s Health, School of Life Course and Population Sciences, Faculty of Life Sciences and Medicine, King’s College London, St Thomas’ Hospital, London, United Kingdom

**Keywords:** Immunology, T cell, activation, neonate, infection, cord blood, heel prick

## Abstract

It is now established that immune maturation occurs along a defined trajectory in the weeks and months after birth, but the immediate changes that occur within immune cells following birth is less clear. In this study, we monitored the immune profile of neonates *via* analysis of paired samples (n= 28) of cord blood and heel prick blood taken at varying times post term delivery by planned elective caesarean section. This paired approach accounted for the between-subject variability often observed over the first week of life. We identified rapid changes in immune cell populations within hours of birth. Specifically, we observed increased proliferation in effector T cells (but not regulatory T cells) that exhibited an increase in cytokine producing ability and also an increase in the percentage of CD3 T cells over this short time frame. This indicates that the mobilisation of the immune system is immediate post birth, presumably as a response to sudden exposure to the external environment, antigen or stress. Hence, immune development may start to occur more rapidly than previously proposed and as such, to study this trajectory, blood sampling should begin as soon after birth as possible.

## Introduction

Sampling from human neonates, particularly healthy infants born at term, is both difficult and ethically challenging. Much data assessing the neonatal immune system is based on the analysis of cells and cytokines detected in cord blood. Whilst there is evidence that suggests cord blood is not representative of post-natal immunity (1), there are few studies that have assessed whether cord blood can be viewed as indicative of the neonatal immune status directly at birth, and moreover, if any important immune changes occur in first few hours post-delivery. There are myriad immunological challenges as neonates transition from a (relatively) sterile intrauterine environment to a non-sterile extrauterine environment, and thus rapid and well-orchestrated processes are required for neonatal survival. Furthermore, there is substantial between-subject variability, even within the first week of life, which makes understanding changes in immune profiles difficult to interpret on a group basis. However, a greater understanding of the immediate immune changes that are evoked after birth are needed to disentangle why cord blood immune parameters sometimes differ from those in neonatal blood and identify those cells that respond to the immediate challenges post birth and how these responses are manifested. This is particularly pertinent within preterm infants, in whom early onset sepsis, due to acquisition of pathogens trans-placentally or *via* ascending infection during the perinatal period, results in mortality rates as high as 29% (2).

Thus, a greater understanding of the transitional immune events post birth may help in both predicting and assisting neonatal response to infection.

## Materials and Methods

**Figure 1:**
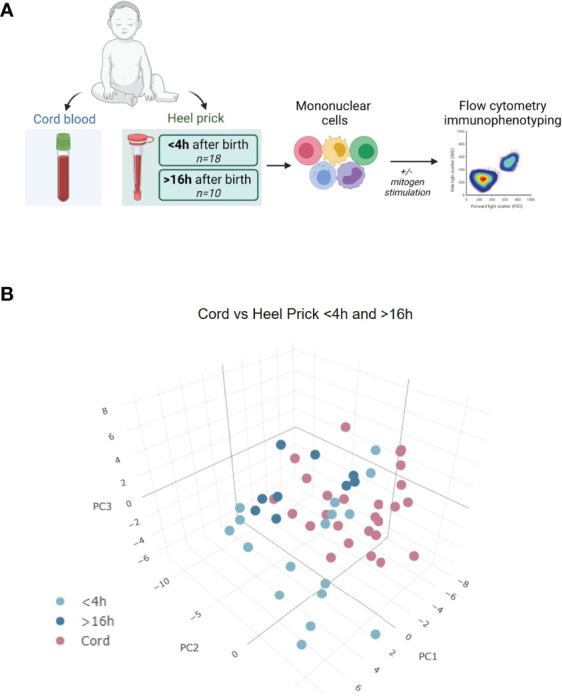
Rapid immune cell changes observed immediately post birth. **(A)** Experimental design: cord blood and heel prick mononuclear cells were collected at birth (cord blood) or within 48 h post birth (heel prick samples). Cells were phenotyped either immediately post thawing or after 4 h polyclonal stimulation, and cytokine responses and activation markers were measured using multiparametric flow cytometry. Created with BioRender.com. **(B)** 3D PCA visualisation showing the distribution of cord blood samples (pink; n=26) vs paired heel prick samples collected within < 4 h of birth (light blue; n=17) or > 16 h of birth (dark blue; n=9) based on 112 parameters determined by flow cytometry (only samples with complete data set included).

Cord blood and paired heel-prick samples were obtained from 28 neonates born between July 2018 and January 2020 (women provided written informed consent as part of the PROMESA study, REC Approval No. 17/LO/0641). All neonates were born at term gestation (≥37 weeks’ gestation) following an elective planned caesarean section (CS) of singleton pregnancies (primiparous or multiparous) of mothers with pre-pregnancy body mass index (BMI) of <35 kg/m^2^ and the women were not in labour. The participant demographic table is presented in **Supplementary Table 1**. Cord blood was collected at birth and subsequently, paired heel prick blood was obtained within 4 h of birth (n=18: median 0.4 h; range, 0.05 – 3.5 h) or between 16 to 48 h post birth (n=10: median, 22 h; range, 17 – 47 h) (protocol described in **Figure 1A**). Mononuclear cells (MCs) were isolated by Ficoll centrifugation from the different blood samples, washed and then frozen in Cryostor^®^ CS10 (Sigma, UK). Samples were analysed in batches by multiparametric flow cytometry. Cord blood mononuclear cells (CBMCs) were thawed and plated in 96 well round bottom plates (Corning, USA) in 200μl of complete media (RPMI [Gibco, UK), 10% FBS, penicillin and streptomycin) with or without stimulation (as detailed in the appropriate figure legends) with phorbol 12-myristate 13-acetate (PMA) (10 ng/ml) (Sigma), Ionomycin (1 μg/ml) (Sigma), Brefeldin A (20 ng/ml) (Sigma) and Monensin solution (2 μM) (BioLegend) for 4 h at 37°C. Cells were stained with one of four antibody panels (**Supplementary Table 2**) assessing different immune populations (with a particular focus on T cell status and function) and analysed by flow cytometry (4-laser LSR Fortessa, BD). Before antibody staining with any of the 4 panels, all cells were stained with Zombie NIR Fixable Viability dye (1:1000 dilution in PBS; BioLegend) for 15 mins 4°C in dark. The TCR Vδ1-FITC antibody (TS8.2; Thermo Fisher) was also added in this step for panel 1. Panels 1-3 were performed on non-stimulated cells stained directly post thawing and panel 4 on cells post the 4 h stimulation (as above). Raw FCS files were analysed using FlowJo (v10.6.2, BD) and the gating strategies are shown in **Supplementary Figure 1**. Cell populations were excluded from downstream analysis if the event count in the parent population was <30. For intracellular staining of cytokines following polyclonal stimulation, the gates were set on the unstimulated (BFA only) control.

Statistical tests: Analysed flow cytometry populations were imported into an Excel spreadsheet and analysed in R (v4.0.3) to generate principal component analysis (PCA) plots, boxplots, paired scatter plots, and linear regression correlation plots. The graph layout for box plots were generated using the geomboxplot function (ggplots package). The corrplot package was used to generate Spearman correlation plots, where the analysis was performed on log-transformed data by Spearman’s rank correlation coefficient. PCA was performed using the PCA methods package analysing 112 immune parameters. For paired data, unadjusted p values (*p<0.05; **p<0.01; ***p<0.001, ****p<0.0001) were assessed using two-sided paired Wilcoxon tests (between paired cord blood and heel prick samples). All correlations non-significant at p > 0.05 level, where lines indicate simple linear regression.

## Results

**Figure 2:**
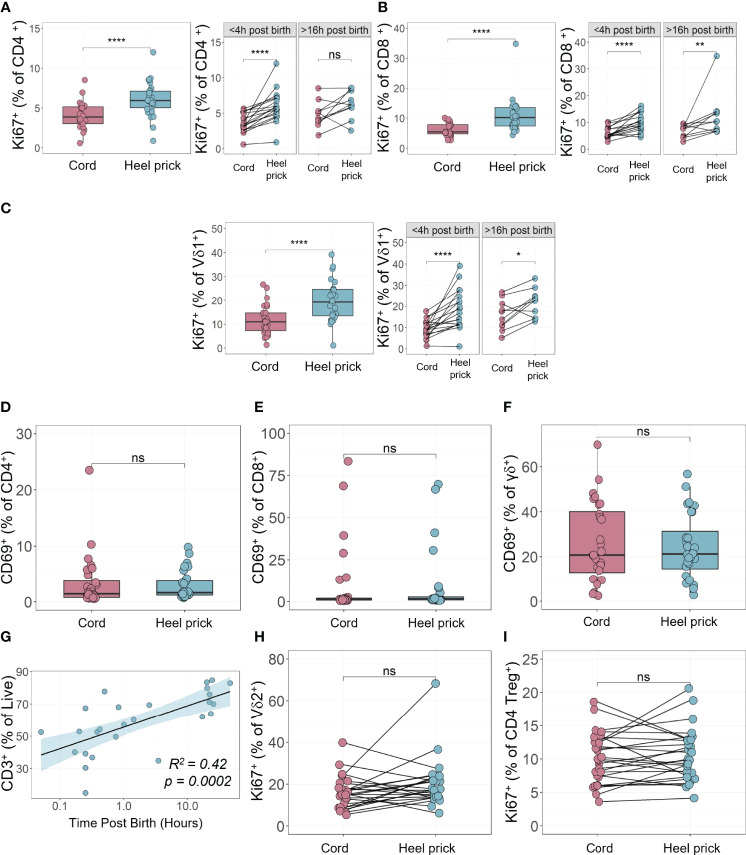
Enhanced proliferation in neonatal T cells observed immediately post birth **(A–C)** The percentage of proliferating (Ki67^+^) CD4^+^ **(A)**, CD8^+^ **(B)**, and Vδ1^+^ γδ **(C)** T cells from unstimulated paired cord blood (n=28) stained immediately post thawing compared to heel prick samples collected <4 h (n=18) or >16 h post birth (n=10) **(D–F)** The percentage of activated (CD69^+^) CD4^+^ **(D)**, CD8^+^ **(E)** and γδ^+^ **(F)** T cells from unstimulated paired cord blood and heel prick samples (n=28) stained immediately post thawing. **(G)** Time dependent linear regression analysis of the percentage of live CD3^+^ T cells from unstimulated heel prick blood samples collected within 48 h post birth (n=28). **(H, I)** The percentage of proliferating (Ki67^+^) Vδ2^+^ γδ **(H)**, and CD4^+^ Treg **(I)** cells from unstimulated paired cord blood and heel prick samples (n=28) stained immediately post thawing. Box and whisker plots follow standard Tukey representations and indicate the median (central line), the interquartile range (upper line = 75^th^ percentile, lower line = 25^th^ percentile), and the whiskers represent 1.5 x 75^th^/25^th^ percentile. P values (NS, non-significant; ****p<0.0001) for all paired data between cord and heel prick samples were assessed using two-sided paired Wilcoxon tests. *p<0.05, **p<0.01.

**Figure 3:**
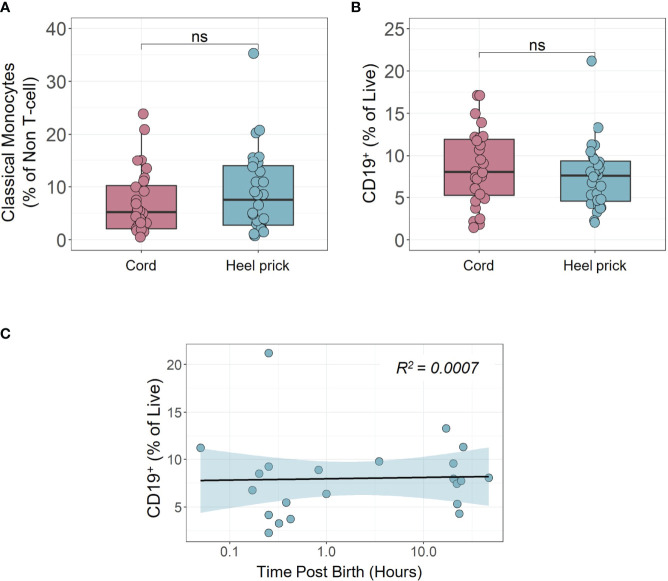
Percentage of neonatal classical monocytes and B cells remain unchanged immediately post birth. Proportion of **(A)** classical monocytes (CMCs) and **(B)** CD19^+^ B cells was measured by multiparametric flow cytometry in unstimulated paired cord blood and heel prick samples (n=28) stained immediately post thawing (n=28). **(C)** Time dependent linear regression analysis of unstimulated CD19^+^ B cells from heel prick blood samples collected within 48 h post birth (n=28). Box and whisker plots follow standard Tukey representations and indicate the median (central line), the interquartile range (upper line = 75^th^ percentile, lower line = 25^th^ percentile), and the whiskers represent 1.5 x 75^th^/25^th^ percentile. NS, non-significant for all paired data between cord and heel prick samples were assessed using two-sided paired Wilcoxon tests. ns, non-significant.

Cord blood was collected after planned CS birth and subsequently, paired heel prick blood was obtained within 4 h of birth (n=18: median 0.4 h; range, 0.05 – 3.5 h) or between 16 to 48 h post birth (n=10: median, 22 h; range, 17 – 47 h) as described in **Figure 1A**. 3D principal component analysis (PCA) was performed, having removed samples that lacked complete data sets, using 112 flow cytometry parameters, that included 33 immune cell types, 28 different cytokine producing cells and a range of other functional immune markers including proliferation as measured by Ki67 staining. Using this unbiased analysis, we compared the immune profile of mononuclear cells isolated from either cord blood or blood samples taken <4 h and >16 h after birth (**Figure 1B**). This analysis showed some sample segregation, suggestive of rapid changes in immune cells within the first 48 h of life. Indeed, even those samples taken within 4 h of birth (n=17, light blue, **Figure 1B**) appeared to segregate from the cord blood samples. We then assessed which of the parameters made the greatest contribution to variations in PC1/PC2. Both cell proliferation and cytokine producing cells appeared within the top ten drivers and so we investigated these parameters further. We observed rapid induction of cell proliferation immediately following birth, in the absence of further stimulation, in several CD3^+^ T cell sub-populations. For example, CD4, CD8 and Vδ1 γδ T cells exhibited significant elevation in the percentage of Ki67^+^ cells within hours of birth (**Figures 2A–C**). This was more apparent in the samples taken within 4 h of birth in contrast to those samples taken later (>16 h post birth). This increased proliferation was not associated with cell activation as determined by equivalent CD69^+^ expression on T cells isolated from cord blood or heel pricks (**Figures 2D–F**). Indeed, we noted that the proportion of CD3^+^ T cells increased with time post birth (p=0.0002, R^2^ = 0.42, **Figure 2G**) consistent with this increased proliferative rate. In comparison, neither Vδ2 γδ T cells nor regulatory T cells (Tregs) showed this immediate enhanced proliferation (**Figures 2H, I**). In contrast to the proliferation and percentage changes observed in these T cell populations, we did not observe any changes in other populations such as classical monocytes (CD16^-^CD14^+^) or B cells between the cord blood and heel prick samples at any time point (**Figures 3A, B**), and there was no correlation with B cells and time post birth (R=0.0007, **Figure 3C**).

**Figure 4:**
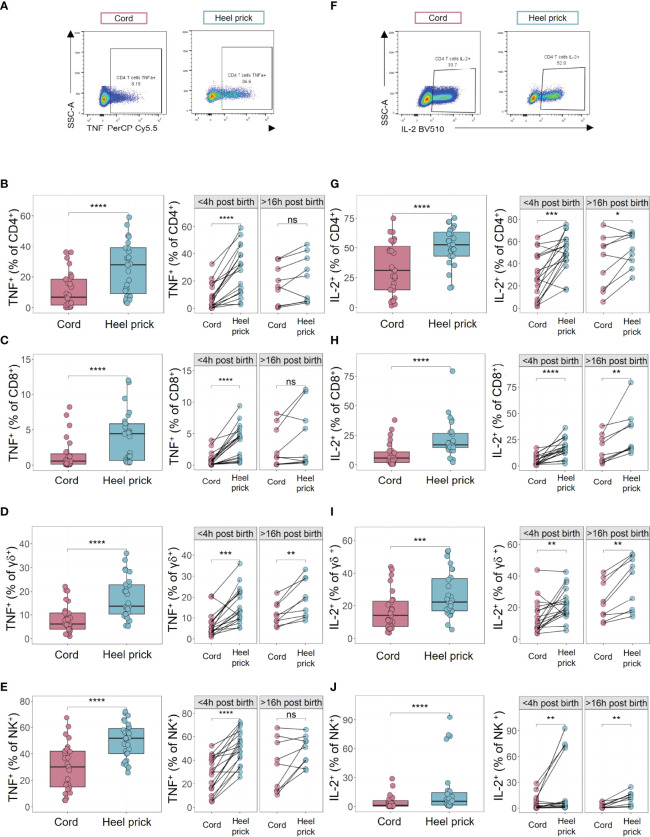
Elevated percentages of cytokine-expressing immune cells observed immediately post birth. Cytokine production was measured by multiparametric flow cytometry in mononuclear cells isolated from cord blood or paired heel prick samples in response to polyclonal stimulation [PMA (10 ng/ml), ionomycin (1 μg/ml), brefeldin A (20 ng/ml) and monensin solution (2 μM) at 37 °C for 4 h]. **(A)** Representative flow plots of TNF in CD4^+^ T cells from cord and heel prick samples. **(B–E)** The percentage of TNF expressing cells in CD4^+^ **(B)**, CD8^+^ **(C)** and γδ^+^ **(D)** and NK **(E)** cells from cord blood (n=28) compared to heel prick samples collected <4 h (n=18) or >16 h post birth (n=10). **(F)** Representative flow plots of IL-2 in CD4^+^ T cells from cord and heel prick samples. **(G–J)** The percentage of IL-2 expressing cells in CD4^+^ **(G)**, CD8^+^ **(H)** and γδ^+^ **(I)** and Natural Killer (NK) **(J)** cells from cord blood (n=28) compared to heel prick samples collected <4 h (n=18) or >16 h post birth (n=10). Box and whisker plots follow standard Tukey representations and indicate the median (central line), the interquartile range (upper line = 75^th^ percentile, lower line = 25^th^ percentile), and the whiskers represent 1.5 x 75^th^/25^th^ percentile. P values (***p<0.001, ****p<0.0001) for all paired data between cord and heel prick samples were assessed using two-sided paired Wilcoxon tests. *p<0.05, **p<0.01, ns, non-significant.

Consistent with a potential mobilisation of the neonatal immune system immediately post birth, we also observed a rapid (< 4 h) increase in the percentage of CD4, CD8, γδ T cells and NK cells producing TNF after *ex vivo* stimulation with PMA/Ionomycin (**Figures 4A–E**). Interestingly, this was not as apparent in stimulated cells isolated from blood > 16 h post birth (**Figures 4A–E**) suggesting a rapid, transitory effect. Consistent with the observed increased proliferation, the percentage of IL-2 producing CD4, CD8, γδ T cells and NK cells was also significantly increased post stimulation in the samples taken within hours of birth (**Figures 4F–J**). The elevated percentages of IL-2-producing cells was still observed in the heel prick samples taken >16 h post birth when compared to their paired cord blood sample, particularly in CD8 and γδ T cells (**Figures 4H, I**). This is consistent with a significantly increased proportion of IL-2-producing CD8 and γδ T cells over time within the heel prick samples (p=0.02, R^2^ = 0.19; p=0.005, R^2^ = 0.26, respectively, **Supplementary Figure 2**).

## Discussion

At birth, the transition from *in utero* to an external environment presents a need for rapid physiological adaptation coupled with an ability to respond to microbial challenges. Whilst it is known that there are conspicuous developmental changes within the first few weeks of life along a very defined trajectory (1, 3, 4) the rapid immune changes we observed in healthy neonates within a few hours of birth have not previously been anticipated. An earlier study reported only 12 differentially expressed genes in transcriptomic analysis of whole blood between day 0 and day 1 although extensive changes thereafter (3). Although some phenotypic cellular changes were also observed in that study, most were reductions (or no change) in normalised cell numbers over the first week of life. Indeed, in our study, despite observing some changes in the percentages of CD3^+^ T cells in this very early time window, the majority of cell types were also unchanged, and most alterations observed were related to the functional state of the different cell types, as opposed to a proportional change in the numbers of different cell populations. Indeed, the similarities between the cord blood and heel prick samples in the basic cell populations e.g. % of CD4, CD8 and ratio of naïve/memory cells argue that these two cellular compartments are equivalent, despite the fact that they represent circulating blood from either the fetal (cord blood) or neonatal (heel prick) circulatory systems. We cannot exclude that potential differences in nutrients or oxygen due to different microvasculature between the two blood sources could be playing a role in the changes observed. Intra-individual heterogeneity precluded using just the heel prick samples over time to assess all changes induced post birth, not least that these were often greatest in the <4 h time frame. Nevertheless, some changes could be observed within just the heel prick samples as seen with CD3 and IL-2 producing T cells. The paired analysis of cord blood and heel prick negated these intra-individual variations allowing changes to be observed. Plasma concentrations of some proteins, such as complement and acute phase proteins are known to increase rapidly post birth with some (e.g., serum amyloid A), showing just a transient rise indicative of an acute response (5). Indeed, much of the changes in both proliferation and cytokine potential observed were not as apparent in samples taken >16 h post birth, suggesting our observations are a transient response to myriad challenges immediately post birth. It is known that several cell types, as well as cytokines, are altered during the natural birth process (6–9). Our samples, however, were all taken after planned caesarean deliveries, and as such, would not have been subjected to the magnitude of hormonal surges or stress response associated with labour and vaginal delivery (10). Indeed, this is a limitation of only studying controls delivering *via* Caesarean section. It is possible that there may have been even greater effects if birth had been preceded by labour but this would also have introduced a lot of individual variation in terms of length of labour. Mothers were excluded from the study if they presented with any infection/pre-eclampsia, conditions that may affect the immune system of the infant (11) and the maternal demographics were very similar for all the participants, representative of mothers in the general population. It should also be noted that maternal immune cells do not differ pre and post caesarean section (12), suggesting we are observing a neonatal specific response to the extrauterine environment. Similarly, whilst proliferating T cells have been observed in neonates born to mothers with chorioamnionitis (4), all neonates in this study were born to healthy mothers with no evidence or record of exposure to placental inflammation or infection.

It is intriguing, therefore, to consider what may be driving this rapid mobilisation of the immune system. Without a comparison to vaginally delivered infants, it is impossible to be sure that the immediate proliferative response is not merely related to the recovery from any anaesthetic used during surgery. Indeed, CS delivered neonates show a heightened behavioural response to stress assessed 2 h after birth (13). Assuming the observed responses are not related solely to mode of delivery, it could be possible that this is this an antigen specific response or a more general response to stress such as cold shock or changes in oxygen tension immediately upon delivery. Environmental temperature has been shown to affect immune parameters in neonatal pigs; where piglets maintained in a cold environment of 18°C and challenged with LPS had greater serum concentrations of TNF and cortisol compared to pigs maintained at 34°C (14). Thus, enhanced TNF as observed, could be related to the cold shock of delivery.

Similarly, could these responses be related to microbial/commensal exposures post birth? Neonatal microbial colonisation begins immediately where the gut microbiotas are highly dynamic and individualised during the neonatal period. This is dependent both on mode of delivery, driven by skin contact of the baby with the mother, and breast feeding (15–17). Also, the disrupted transmission of the maternal gastrointestinal bacteria observed in C-section babies associates with a substantially higher relative abundance of opportunistic pathogens commonly associated with the healthcare and hospital environment (18) which may potentially drive greater responses. This exposure to microbial antigens facilitates the development and maturation of the neonatal immune system (19). Hence, this initial rapid colonisation may be at least initiating the responses observed.

The increase in IL-2-producing cells would be consistent with the enhanced proliferation of T cells for which IL-2 is an autocrine growth factor, induced downstream of T cell activation. This notwithstanding, we did not observe any enhanced proliferation in Tregs which are usually more responsive to IL-2 stimulation. Indeed, the specific induction of effector (but not regulatory) T cell proliferation may facilitate an immediate effector T cell response to multiple antigen exposure without inhibition. This study did not assess T cell clonality, however, and hence we could not establish whether this is a specific T cell receptor mediated response to antigen. Whilst the increase in proliferation observed was rapid, it must be noted that Ki67 expression, a marker extensively used to indicate cell proliferation, only delineates cells that are not in G_0_ (20) and how long these cells remain in, or indeed if they exit G_1_, cannot be elucidated. Similarly, not all cells showed an increase in Ki67 immediately post birth. Notably, the percentage of B cells remained constant, despite these cell populations being known to expand at least in the first few months post birth (4).

Our study suggests that the neonatal immune system rapidly changes within the first few hours of birth as the neonate transitions from the protected *in utero* environment to the diverse microbial environment. This has broad implications for a better understanding of how the neonate protects itself in these early hours/days and how this could be enhanced therapeutically. Our study highlights a greater need to focus on this very early development state of the human neonatal immune system.

## Data Availability Statement

All FCS files, from which the data presented has been generated, have been deposited in the Flow Repository: http://flowrepository.org/id/FR-FCM-Z524. Any further inquiries can be directed to the corresponding author.

## Ethics Statement

The human participants were recruited as part of the PROMESA study, which was reviewed and approved with REC Approval No. 17/LO/0641. Written informed consent to participate in this study was provided by the participants’ legal guardian/next of kin.

## Author Contributions

GA: Flow cytometry analysis and manuscript drafting. SG, SK: Sample processing, flow cytometry and analysis. SK, AD: panel design, flow cytometry. DG and RT: Study conception, experimental design and manuscript drafting. All authors reviewed drafts of the manuscript prior to submission. All authors contributed to the article and approved the submitted version.

## Funding

GA is supported by Action Medical Research grant held by DG (GN2790); SG is supported by a MRC-KCL Doctoral Training Partnership in Biomedical Sciences (MR/N013700/1), RT by Tommy’s (Charity No. 1060508) and Borne (1167073). The research was also supported by the National Institute for Health Research (NIHR) Biomedical Research Centre based at Guy’s and St Thomas’ NHS Foundation Trust (GSTT) and King’s College London (KCL) (part of the King’s Health Partners Academic Sciences Centre).

## Author Disclaimer

The views expressed are those of the authors and not necessarily those of the NHS, the NIHR or the Department of Health.

## Conflict of Interest

The authors declare that this study received funding from Evolve Biosystems (RT and DG). The funder had the following involvement with the study: neonatal cord blood and heel prick blood collection.

## Publisher’s Note

All claims expressed in this article are solely those of the authors and do not necessarily represent those of their affiliated organizations, or those of the publisher, the editors and the reviewers. Any product that may be evaluated in this article, or claim that may be made by its manufacturer, is not guaranteed or endorsed by the publisher.
