# Supplementary material for: Activation of Lymphocytes in Healthy Neonates Within Hours of Birth
Source: Front Immunol. 2022 May 31;13:883933. doi: 10.3389/fimmu.2022.883933 (PMC9195076; doi:10.3389/fimmu.2022.883933)
Supplement: Supplementary file 1 [file DataSheet_1.zip › Supplementary Tables.docx]

Supplementary Table 1. **Demographic table of participants**

| **Subjects** |  | n=28 |
| --- | --- | --- |
| **Median (range) maternal age at recruitment** | 35.5 (28 to 46 years) |  |
| **Ethnicity** | White (British, Irish, Arab, European, African) | 23 |
|  | Black (African, Caribbean, Black British) | 3 |
|  | Other (South/Latin American) | 2 |
| **Current underlying conditions** | Asthma | 2 |
|  | Gestational Diabetes | 2 |
|  | Eczema/atopic dermatitis | 3 |
|  | Hyper/hypo thyroidism | 2 |
|  | Gastritis | 1 |
|  | Cardiac | 1 |
| **Reason for CS** | Previous CS | 9 |
|  | Malpresentation | 5 |
|  | Other (tocophobia/previous traumatic birth/maternal age/placenta praevia/large baby etc.) | 13 |
|  | Unknown | 1 |

Supplementary Table 2. **Flow cytometry panels**

| **Panel 1** | | | | | |
| --- | --- | --- | --- | --- | --- |
| **Marker** | **Fluorophore** | **Supplier** | **Cat No.** | **Clone** | **Dilution** |
| CD45RA | BV786 | Biolegend | 304139 | HI 100 | 1/100 |
| CCR7 | BV650 | Biolegend | 353233 | GO43H7 | 1/50 |
| CD8α | BV510 | Biolegend | 301047 | RPA-T8 | 1/400 |
| Ki-67 | BV421 | Biolegend | 350505 | Ki-67 | 1/50 |
| CD4 | PE-Cy7 | Biolegend | 317413 | OKt4 | 1/50 |
| CD25 | PE | Biolegend | 356103 | MA-251 | 1/50 |
| TCR Vδ2 | PerCP | Biolegend | 331410 | B6 | 1/50 |
| TCR Vδ1 | FITC | Thermo Scientific | TCR2730 | TS8.2 | 1/100 |
| FOXP3 | APC | Biolegend | 320214 | 259 d | 1/50 |
| CD3 | AF700 | Biolegend | 317339 | OKT3 | 1/200 |
| Human TruStain Fcx | N/A | Biolegend | 422392 | N/A | 2.5/50 |
| **Panel 2** | | | | | |
| **Marker** | **Fluorophore** | **Supplier** | **Cat No.** | **Clone** | **Dilution** |
| HLA-DR | BV786 | Biolegend | 307642 | L243 | 1/100 |
| CD11c | BV650 | Biolegend | 301637 | 3.9 | 1/50 |
| CD40 | BV510 | Biolegend | 334329 | 5c3 | 1/100 |
| CD14 | BV421 | Biolegend | 301829 | M5E2 | 1/100 |
| CD16 | PE-Cy7 | Biolegend | 302015 | 3G9 | 1/200 |
| CD19 | PE Dazzle | Biolegend | 302252 | HIB 19 | 1/100 |
| CD86 | PE | Biolegend | 305405 | IT2.2 | 1/100 |
| CD123 | PerCP Cy5.5 | Biolegend | 306015 | 6H6 | 1/50 |
| CD303 | FITC | Biolegend | 307619 | L243 | 1/100 |
| CD1c(BDCA-1) | APC | Biolegend | 331523 | L161 | 1/100 |
| CD3 | AF700 | Biolegend | 317339 | OKT3 | 1/200 |
| Human TruStain Fcx | N/A | Biolegend | 422392 | N/A | 2.5/50 |
| **Panel 3** | | | | | |
| **Marker** | **Fluorophore** | **Supplier** | **Cat No.** | **Clone** | **Dilution** |
| CD4 | BV786 | Biolegend | 317442 | Okt4 | 1/50 |
| CD38 | BV650 | Biolegend | 356619 | HB-7 | 1/100 |
| CD8 | BV510 | Biolegend | 301047 | RPA-T8 | 1/400 |
| CD56 | BV421 | Biolegend | 362551 | 5.1h11 | 1/200 |
| TCR γδ | PE-Cy7 | Biolegend | 331222 | B1 | 1/50 |
| CD69 | PerCP-Cy5.5 | Biolegend | 310925 | FN50 | 1/50 |
| CD161 | FITC | Biolegend | 339923 | HP-3G10 | 1/50 |
| NKG2D | APC | Biolegend | 320807 | 1D11 | 1/50 |
| CD3 | Af700 | Biolegend | 317339 | OKT3 | 1/200 |
| CD16 | APC CY7 | Biolegend | 302018 | 3G8 | 1/100 |
| CD14 | APC CY7 | Biolegend | 325620 | HCD14 | 1/100 |
| Human TruStain Fcx | N/A | Biolegend | 422392 | N/A | 2.5/50 |

| **Panel 4** | | | | | |
| --- | --- | --- | --- | --- | --- |
| **Marker** | **Fluorophore** | **Supplier** | **Cat No.** | **Clone** | **Dilution** |
| CD4 | BV786 | Biolegend | 317442 | Okt4 | 1/50 |
| IFNγ | BV650 | Biolegend | 502538 | 4S.B3 | 1/50 |
| CD8a | BV605 | Biolegend | 301040 | RPA-T8 | 1/100 |
| IL-2 | BV510 | Biolegend | 500338 | MQ1-17H12 | 1/50 |
| CD56 | BV421 | Biolegend | 362551 | 5.1h11 | 1/200 |
| TCR γδ | PECY7 | Biolegend | 331222 | B1 | 1/50 |
| IL-10 | PE Dazzle | Biolegend | 501426 | JES3-9D7 | 1/50 |
| IL-4 | PE | Biolegend | 500705 | 8D4-8 | 1/50 |
| TNF | PErCP Cy5.5 | Biolegend | 502926 | MAb11 | 1/50 |
| CXCL8 | FITC | Biolegend | 511406 | E8N1 | 1/50 |
| CD3 | AF700 | Biolegend | 317339 | OKT3 | 1/200 |
| IL17A | AF647 | Biolegend | 512310 | BL168 | 1/50 |
| IL17F | AF647 | BD | 561333 | 033-782 | 1/50 |
