# Supplementary figures and images for: Activation of Lymphocytes in Healthy Neonates Within Hours of Birth
Source: Front Immunol. 2022 May 31;13:883933. doi: 10.3389/fimmu.2022.883933 (PMC9195076; doi:10.3389/fimmu.2022.883933)

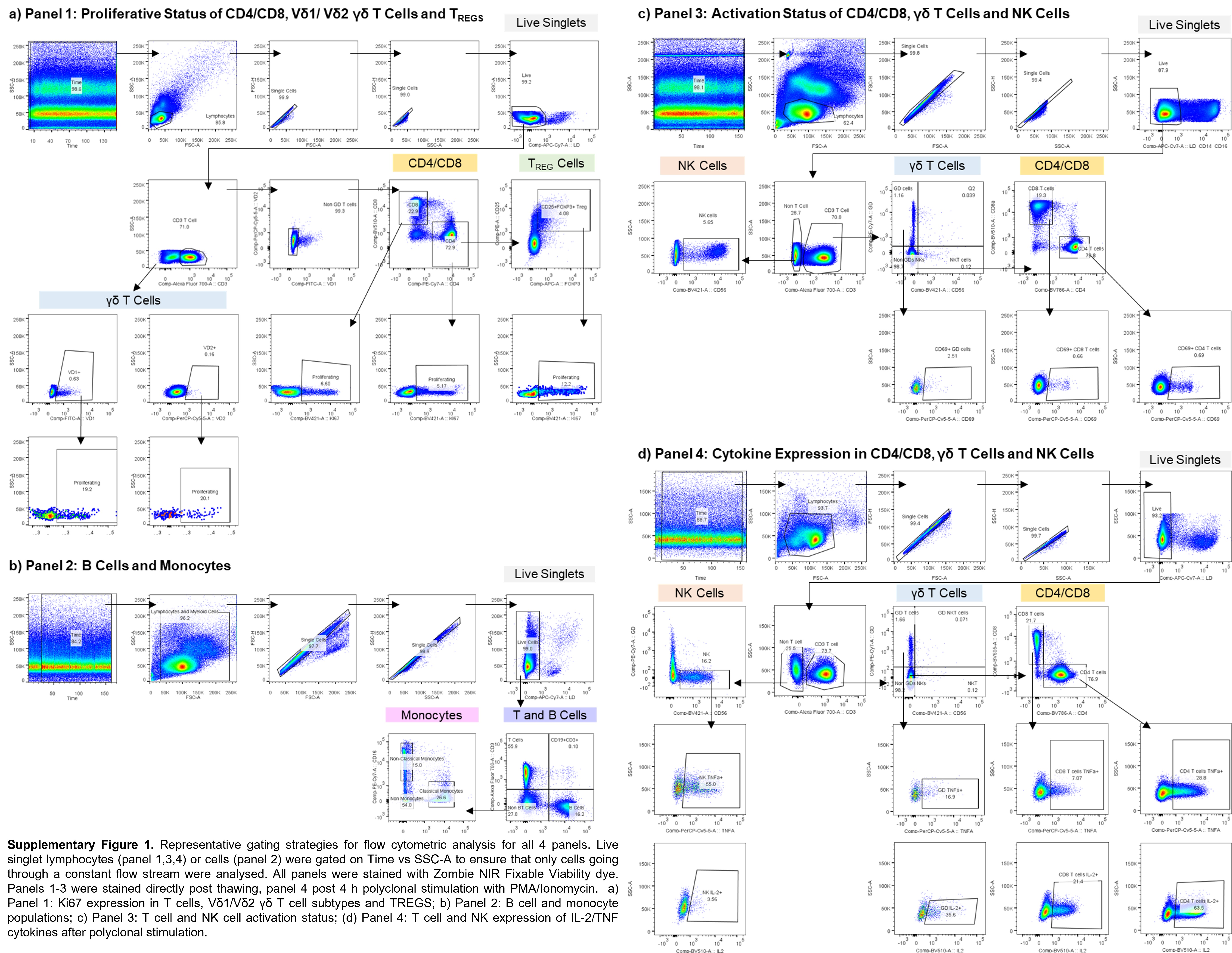

Supplement: Supplementary file 1 [file DataSheet_1.zip › Supplementary Figure 1.jpg]

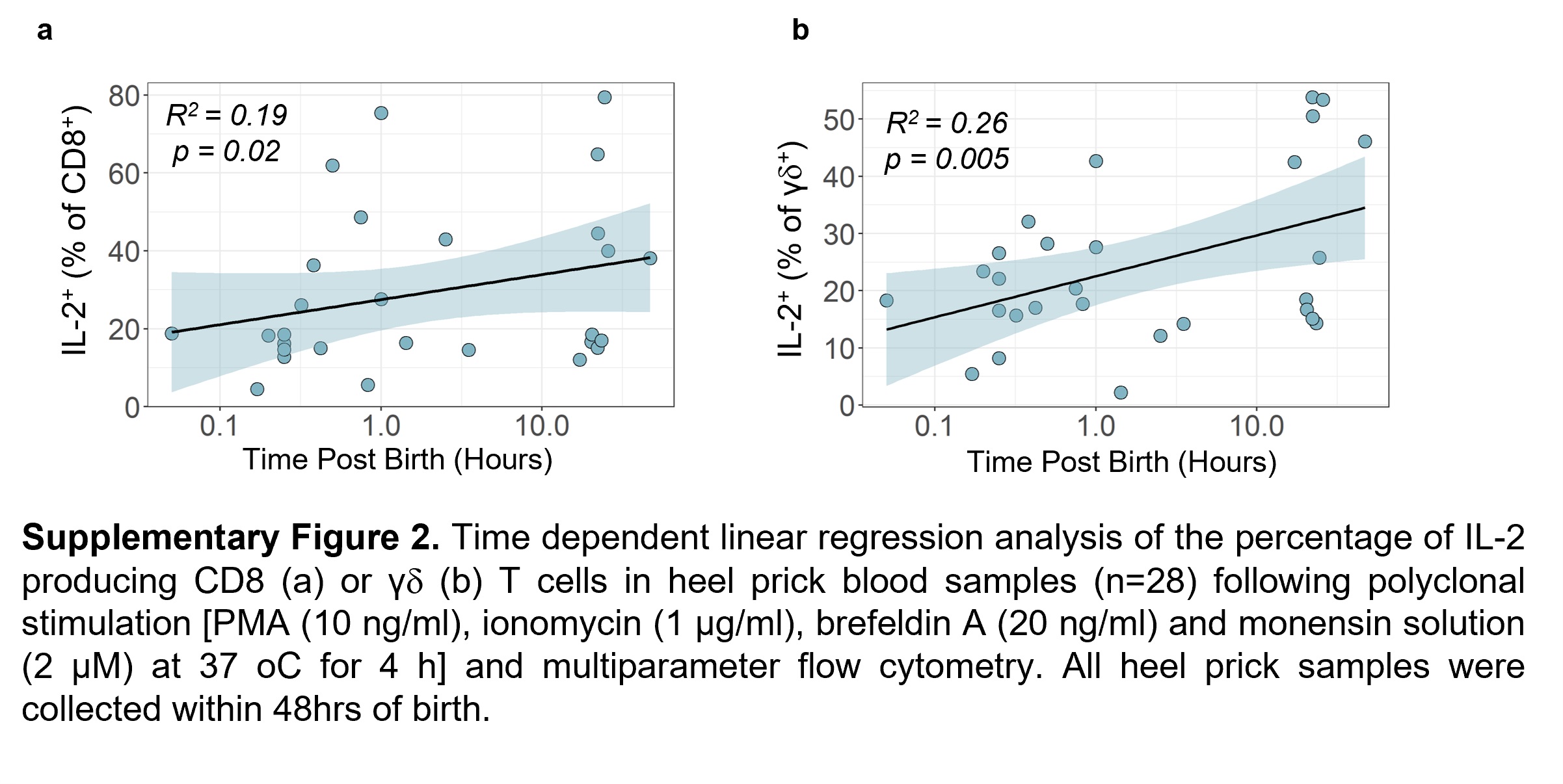

Supplement: Supplementary file 1 [file DataSheet_1.zip › Supplementary Figure 2.jpg]
